# Supplementary figures and images for: Influence of Systemic Therapy on the Expression and Activity of Selected STAT Proteins in Prostate Cancer Tissue
Source: Life (Basel). 2022 Feb 6;12(2):240. doi: 10.3390/life12020240 (PMC8877682; doi:10.3390/life12020240)

Figure S1

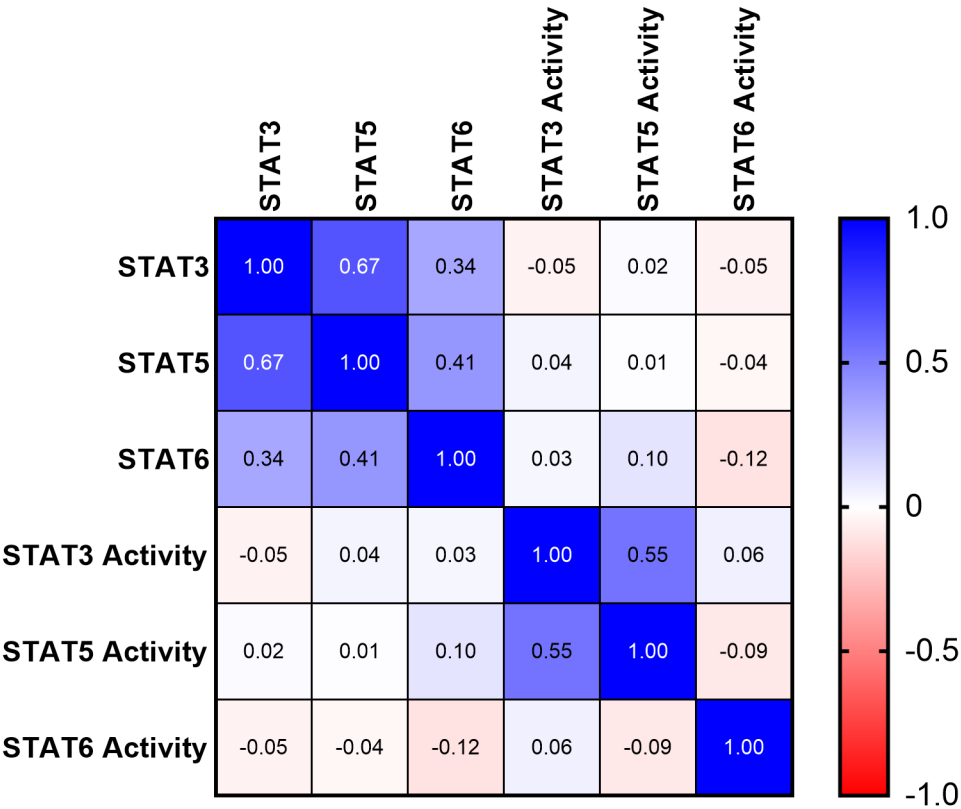

Figure S2

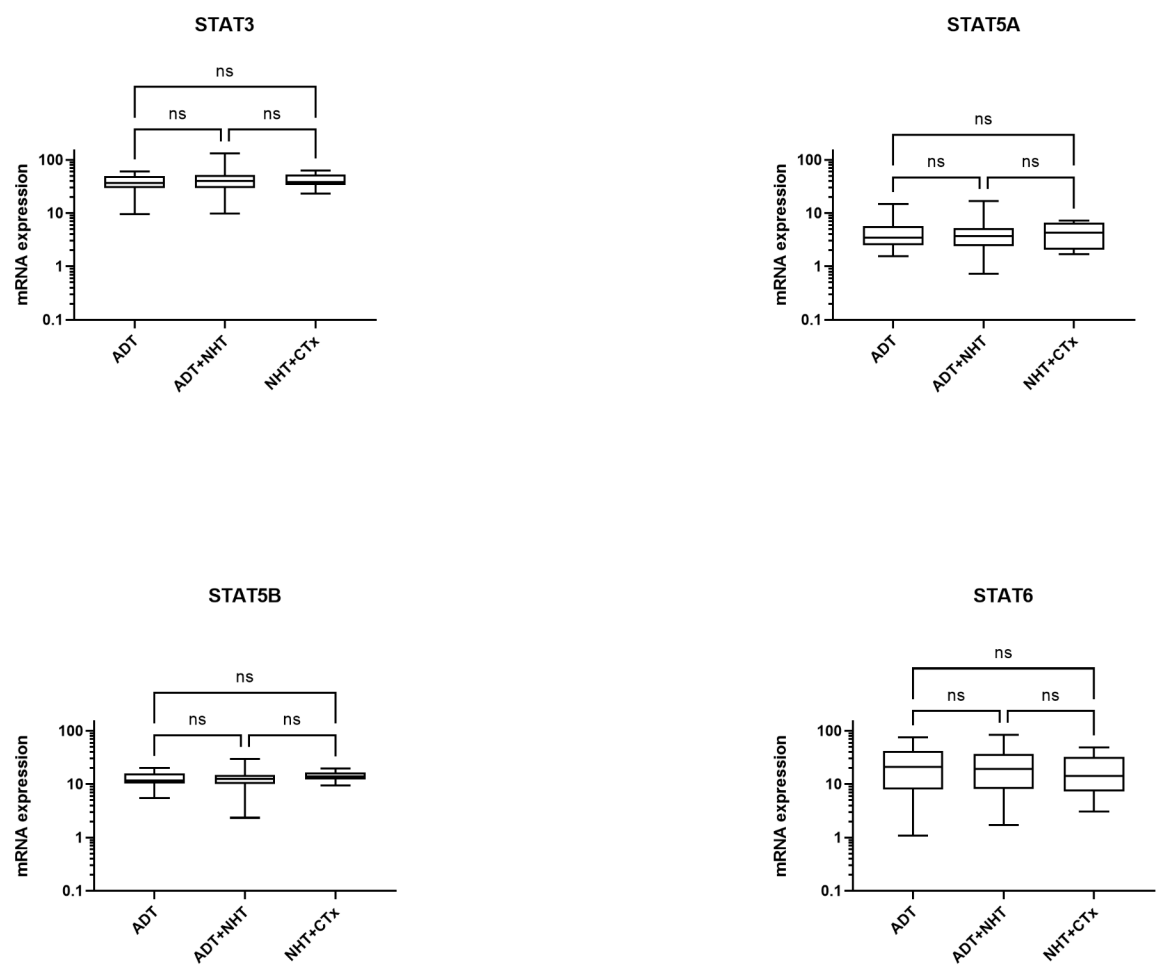

Supplement: Supplementary file 1 [file life-12-00240-s001.zip › life-1579891-supplementary.pdf]
